# The Effects of Levosimendan Exposure on Oxidant/Antioxidant Status and Trace Element Levels in the Pulmonary Artery of Rats

**DOI:** 10.1007/s00232-013-9559-2

**Published:** 2013-05-15

**Authors:** Yasin Ay, Cemalettin Aydın, Halil Basel, Hava Bektaş, Gülay Bülüt, Bekir İnan, Nuray Kahraman Ay, İsmail Demir

**Affiliations:** 1 Department of Cardiovascular Surgery, Bezmi Alem Vakif University Hospital, Istanbul, 64000 Turkey; 2 Department of Biophysics, Yuzuncu Yıl University, Van, Turkey; 3 Department of Pathology, Yuzuncu Yıl University, Van, Turkey; 4 Department of Cardiology, Bezmi Alem Vakif University Hospital, Istanbul, Turkey; 5 Department of Cardiovascular Surgery, Kosuyolu Heart Training and Research Hospital, Istanbul, Turkey

**Keywords:** Levosimendan, Oxidative stress, Free radical, Oxidant/antioxidant, Pulmonary artery

## Abstract

We investigated both the effect of levosimendan and the role of oxidant/antioxidant status and trace element levels in the pulmonary artery of rats. Fourteen male Wistar albino rats were randomly divided into two groups of seven animals each. Group 1 was not exposed to levosimendan and served as a control. Levosimendan (12 μg/kg) diluted in 10 ml 0.9 % NaCl was administered intraperitoneally to group 2. Animals of both groups were killed after 3 days, and their pulmonary arteries were harvested to determine changes in tissue oxidant/antioxidant status and trace element levels. The animals in both groups were killed 72 h after the levosimendan exposure treatment, and pulmonary arteries were harvested to determine levels of the lipid peroxidation product MDA and the antioxidant GSH as well as the decreased activity of antioxidant enzymes such as SOD, GSH-Px and CAT. It was found that MDA levels increased in pulmonary artery tissues of rats after levosimendan administration. The GSH level decreased in the pulmonary artery of rats after levosimendan treatment. Co, Mn, Fe, Cd and Pb levels were significantly higher (*P* < 0.001) and Mg, Zn and Cu levels significantly lower (*P* < 0.001) in the levosimendan group compared to the control group. These results suggest that levosimendan treatment caused an increase in free radical production and a decrease in antioxidant enzyme activity in the pulmonary artery of levosimendan-treated rats. It also caused a decrease or increase in the levels of many minerals in the pulmonary artery, which is an undesirable condition for normal pharmacological function.

## Introduction

Levosimendan causes venous, arterial and coronary vasodilation, probably by opening ATP-sensitive potassium channels in smooth muscle. Dose-dependent hypotension may occur. Levosimendan is also of benefit in the setting of pulmonary vasoconstriction and right ventricular dysfunction and reduces pulmonary vascular resistance (Kamath et al. 2009). Congestive heart failure is a complex hemodynamic and metabolic syndrome in which both cardiac function and metabolism are impaired (Cohen-Solal et al. 1995). An important characteristic of this condition is an imbalance between energy production and utilization. Oxygen consumption in the heart is correlated with cardiac workload (Balaban 1990). Dilated cardiomyopathy refers to a weakened cardiac muscle that is unable to pump strongly enough to empty the heart properly in each beat. In restrictive cardiomyopathy, the muscle becomes so stiffened and inextensible that the heart cannot fill properly between beats. The degeneration of myocardium associated with muscular dystrophy is often accompanied by cardiomyopathy (Colledge et al. 2010; Basel et al. 2012). Levosimendan is a cardiovascular drug for the treatment of acute and decompensated heart failure. It has positive inotropic and antistunning effects mediated by calcium sensitization of contractile proteins (Pollesello and Papp 2007; Jamali et al. 1997) and vasodilatory and anti-ischemic effects mediated by the opening of ATP-sensitive potassium (KATP) channels in vascular smooth muscle cells (Jamali et al. 1997; De Witt et al. 2002). Levosimendan has a vasodilatory effect via opening of KATP channels in the plasma membrane of vascular smooth muscle cells, activates myocardial mitochondrial KATP (m-KATP) channels and exerts a PreC effect against ischemia–reperfusion injury (Kersten et al. 2000).

The family of reactive oxygen species (ROS) includes highly bioactive, short-living molecules that are derived from reduction of molecular oxygen. Multiple enzyme systems use different substrates as sources of electrons to produce a variety of ROS, including superoxide, hydroxyl radical, hydrogen peroxide, peroxynitrite, hypochlorous acid and lipid radicals. Several enzymes are expressed in vascular tissue that contribute to the production as well as to the degradation of ROS. Under physiological conditions, ROS formation and elimination are delicately balanced in the vascular wall. However, enhanced activity of oxidant enzymes and/or reduced activity of antioxidant enzymes lead to oxidative stress (Wassmann et al. 2004). The superoxide dismutases (SODs) are a major cellular defense system against superoxide in all vascular cells. These enzymes contain redox metals in the catalytic center and dismutase superoxide radicals to hydrogen peroxide and oxygen (Wassmann et al. 2004). Reduced glutathione (GSH) plays a major role in the regulation of the intracellular redox state of vascular cells by providing reducing equivalents for many biochemical pathways. Glutathione peroxidase (GPx) is a selenium-containing antioxidant enzyme that effectively reduces hydrogen peroxide and lipid peroxides to water and lipid alcohols, respectively, and in turn oxidizes glutathione to glutathione disulfide. Malondialdehyde (MDA) is an indicator of lipid peroxidation and increases in various diseases (Nordberg and Arner 2001). Catalase (CAT) is mainly a heme-containing enzyme. The predominant subcellular localization of enzyme is peroxisomes, in which it catalyzes the dismutation of hydrogen peroxide to water and molecular oxygen. CAT activity is highest in the liver, relatively high in kidney and very low in heart (Nordberg and Arner 2001).

Trace elements are being increasingly recognized as essential mediators for the development and progression of heart diseases. It is well known that selenium (Se), zinc (Zn) and copper (Cu) in serum can affect certain heart diseases such as Keshan disease heart failure, cardiomyopathy and atherosclerosis. Therefore, trace elements may play an important role in the etiopathogenesis of the diseases (Kosar et al. 2006). Trace elements may also play an important role in alleviating tissue damage due to formation of free radicals.

Hence, a systemic approach has been used in the present study to focus on cardiovascular disease. Oxidative stress was measured by the serum levels of MDA, enzymic GPx and SOD as well as trace element levels in the pulmonary artery. With the assumption that levosimendan directed to the pulmonary artery also affects adjacent organs in rats, we designed our study to investigate the effect of levosimendan exposure of the pulmonary artery on oxidative stress and some trace element levels in the pulmonary artery.

## Materials and Methods

### Treatment of Animals

Fourteen male Wistar albino rats, approximately 6 months of age, with an average body weight of 250–300 g were obtained from the Animal Laboratory of Bezmialem Vakif University (Istanbul, Turkey). Rats were housed in specific cages (18 rats/5 rats). A 12-h light/dark cycle was maintained, and the rats were fed ad libitum.

The animals were randomly divided into two groups, each consisting of seven rats. The animals in group 1 were not treated with drug and served as a control. Levosimendan (12 μg/kg) diluted in 10 ml 0.9 % NaCl was administered intraperitoneally. After 3 days, animals in both groups were killed and their pulmonary arteries harvested for the evaluation of tissue oxidant/antioxidant status and trace element levels after levosimendan exposure.

### Biochemical Analysis

#### Measurement of MDA Level

A 50-mg tissue specimen was homogenized in 0.15 M KCl. After centrifugation of the homogenate at 1,600×*g*, MDA levels in tissue homogenate supernatant were determined by the thiobarbituric acid (TBA) reaction according to Yagi (1994). The principle of this method is based on measuring absorbance of the pink color produced by the interaction of TBA with MDA at 530 nm.

#### Measurements of SOD and GSH-Px Enzyme Activities

Tissues were homogenized in physiological saline (1 g in 5 ml) using a homogenizer (B. Braun, Melsungen, Germany) and centrifuged at 4,000×*g* for 20 min (Heraus, Hanau, Germany). GSH-Px activity was determined by monitoring the changes in NADPH absorbance at 340 nm and by measuring the decrease of H_2_O_2_ absorbance at 240 nm (Aebi 1974). SOD activity was measured by the method based on the nitroblue tetrazolium (NBT) reduction rate. One unit of SOD activity is the amount of enzyme protein causing 50 % inhibition of the NBT reduction rate (Durak et al. 1996).

#### Measurement of GSH Level

GSH levels were measured by the technique of Sedlak and Lindsay (1968) at 412 nm. Samples were precipitated with 50 % trichloroethanoic acid and centrifuged at 1,000×*g* for 5 min. The reaction mixture contained 0.5 ml of supernatant, 2.0 ml of Tris-EDTA buffer (0.2 M, pH 8.9) and 0.1 ml of 10 mM dithiobis(2-nitrobenzoic acid) (DTNB). The solution was kept at room temperature for 5 min, and subsequently absorbance was read at 412 nm.

#### Measurement of CAT Enzyme Activity

Erythrocyte CAT activity was measured by the method described by Aebi (1974). Briefly, the supernatant (0.1 ml) was added to a quartz cuvette containing 2.95 ml of 19 mM H_2_O_2_ prepared in potassium phosphate buffer (50 mM, pH 7.00). The change in absorbance was monitored at 240 nm for 5 min using a spectrophotometer (UV-1201; Shimadzu, Kyoto, Japan).

#### Measurement of Carbonic Anhydrase Activity

Carbonic anhydrase (CA) activity was assayed by hydration of CO_2_ measured by the method of Rickli and Wilbur-Anderson (Topcuoglu et al. 2009) using bromothymol blue as indicator.

#### Measurements of Mineral Levels

Two milliliters of the mixture of HNO_3_/H_2_O_2_ (2:1) was added to 0.7 g of the tissue sample. The mixture was placed in a water bath at 70 °C for 30 min and stirred occasionally; subsequently, 1.0 ml of the same acid mixture was added and the suspension was transferred to a Teflon vessel for digestion in a microwave oven. The bomb was closed, and radiation was applied for 3 min at 450 W. After the addition of 0.5 ml of the same acid mixture, radiation was repeated for 3 min. After cooling for 5 min, 2.0 ml of 0.1 M HNO_3_ was added, and the solution was transferred to a Pyrex tube. After centrifugation, clear solution was used for the determination of Cu, Zn, Mg, Mn, Pb, Cd and Fe (Bush et al. 1995). Measurements were performed by atomic absorption spectrophotometry using a UNICAM-929 spectrophotometer (Unicam, Leeds, UK).

### Statistical Analysis

Data are reported as means ± SD. The parameters were compared between two groups using the Mann–Whitney *U* test. All statistical analyses were carried out using the SPSS^®^ statistical software package (SPSS for Windows version 13.0; SPSS, Inc., Chicago, IL, USA), and *P* ≤ 0.05 was considered significant.

## Results

The lipid peroxidation product MDA level increased significantly (*P* < 0.001) in the pulmonary artery of rats after levosimendan treatment (Table 1). The level of the antioxidant GSH decreased in the pulmonary artery of rats after levosimendan treatment. Co, Mn, Fe, Cd and Pb levels in the pulmonary artery were significantly higher (*P* < 0.001); and Mg, Zn and Cu were significantly decreased (*P* < 0.001) in the levosimendan group compared to the control group (Table 2).

**Table 1:** Effect of levosimendan administration on tissue pulmonary artery levels of MDA, an indicator of oxidative stress, and levels of enzymes that act in cell defense against oxidative stress

	Control			Levosimendan			
	Median	Mean	SD	Median	Mean	SD	*P*
SOD (U/mg)	18.980	19.046	0.380	8.183	7.946*	0.890	0.001
MDA (mg/dl)	86.051	85.883	0.580	116.785	117.192*	1.388	0.001
GSH-Px (EU/gHb)^−1^	141.100	141.614	3.268	77.602	77.502*	0.357	0.001
GSH (EU/gHb)^−1^	104.045	103.734	3.706	77.355	77.417*	0.385	0.001
CA (EU/gHb)^−1^	0.066	0.067	0.003	0.077	0.070	0.012	0.639
CAT (EU/gHb)^−1^	62.111	62.419	1.536	41.643	41.714*	2.868	0.001

Data are reported as mean ± SD for *n* = 8/group. Rats were treated with levosimendan (12 μg/kg day, ip) for 3 days

*SOD* superoxide dismutase, *GSH-Px* glutathione peroxidase, *CA* carbonic anhydrase, *CAT* catalase, *GSH* antioxidant glutathione, *MDA* malondialdehyde

* P < 0.001 compared to control

**Table 2:** Effect of levosimendan treatment on pulmonary artery tissue trace element levels

	Control			Levosimendan			
	Median	Mean	SD	Median	Mean	SD	*P*
Co (μg/dl)	0.322	0.325	0.014	0.552	0.568*	0.044	0.001
Pb (μg/dl)	0.083	0.072	0.022	0.270	0.280*	0.050	0.001
Cd (μg/dl)	0.014	0.015	0.002	0.076	0.076*	0.004	0.001
Mg (μg/dl)	28.550	28.344	0.616	18.560	18.849*	1.165	0.001
Mn (μg/dl)	0.014	0.013	0.002	0.054	0.054*	0.004	0.001
Fe (μg/dl)	1.460	1.427	0.088	30.470	30.350*	0.406	0.001
Cu (μg/dl)	0.122	0.126	0.022	0.075	0.074*	0.003	0.001
Zn (μg/dl)	4.855	4.787	0.516	3.475	3.255*	0.500	0.001

Data are reported as mean ± SD for *n* = 10/group. Rats were treated with levosimendan (12 μg/kg day, ip) for 3 days

* P < 0.001 compared to control

## Discussion

Atherosclerosis is a process for which there is substantial evidence of a role for oxidative stress. Hypercholesterolemia and triglyceridemia are independent risk factors that alone or together can accelerate the development of coronary artery disease and progression of atherosclerotic lesions (Kaviarasan et al. 2005). This study was carried out to investigate the effect of intraperitoneal injection of levosimendan on oxidative stress of pulmonary artery in rats. The pharmacokinetics of levosimendan underpins the prolonged beneficial hemodynamic effects that result from a single-dose regime. It is an appealing and promising alternative adjunct to current therapies for cardiac failure. Levosimendan is also of benefit in the setting of pulmonary vasoconstriction and right ventricular dysfunction (Kamath et al. 2009). Levosimendan also has been evaluated as a bridge therapy for the perioperative phase of cardiac surgery (Pollesello and Papp 2007). Many compounds that open KATP channels in the cell plasma membrane also have been shown to act on KATP channels in mitochondria (Grover and Garlid 2000; Gross 2000). There is a consensus that the opening of m-KATP channels (Gross 2000) protects the heart against ischemia–reperfusion damage. The increased potassium influx associated with m-KATP channel opening is sufficient to protect/preserve mitochondrial function, most probably via the normalization of matrix and intermembrane space volumes, in situations of distress such as ischemia and/or reperfusion (Garlid et al. 2003).

In the present study, it was found that lipid peroxidation products decreased in the pulmonary artery after levosimendan treatment in rats. These results suggest that levosimendan administration may increase or decrease oxidative stress by decreasing free radical production in the pulmonary artery. Oxidative stress may lead to many cellular events, such as inactivation of NO, oxidative modifications of DNA and proteins, lipid oxidation, enhanced mitogenicity and apoptosis of vascular cells as well as increased expression and activation of redox-sensitive genes, such as the receptor for oxidized LDL, adhesion molecules, chemotaxis factors, proinflammatory cytokines, regulators of cell cycle progression and matrix metalloproteinases. Several cardiovascular risk factors are associated with oxidative stress and dysregulated expression and activity of oxidant and antioxidant enzymes, including arterial hypertension and hypercholesterolemia; and the described effects of ROS and impaired NO bioactivity in vascular cells contribute to the development and progression of atherosclerosis at all stages of the disease. Increased levels of ROS were demonstrated in all layers of the diseased arterial wall and within the atherosclerotic plaque. Many studies have demonstrated upregulation of several vascular NADPH oxidase subunits and increased enzyme activity in atherosclerotic animals and humans and after vascular injury (Sorescu et al. 2002; Spiekermann et al. 2003; Szöcs et al. 2002).

The antioxidant defense systems exist to prevent the formation of these increased reactive and free radicals. These include SOD, CA and other free radical scavengers, such as GSH. In our study, GSH levels increased and the activities of antioxidant enzymes, such as SOD and CA, decreased in pulmonary artery tissues of levosimendan-treated rats. The increased formation of ROS and decreased antioxidant defense are defined as oxidative stress, which is widely recognized as an important feature of many diseases. The decrease in antioxidant enzyme activities might be due to their use against free radical destruction or their inhibition by free radical species (Percy et al. 1990). DNA and proteins cause a loss of cell integrity, enzyme function and genomic stability (Paglia and Valentine 1967). Because glucose-6-phosphate dehydrogenase (G6PD) catalyzes the first step of the pentose phosphate pathway, which provides the NADPH necessary for conversion of oxidized glutathione to GSH, the increased tissue GSH level was probably due to the increased G6PD activity that caused the increased production of GSH.

A healthy life depends on the existence of appropriate amounts of various heavy elements. Deficiency in any of these trace elements leads to undesirable pathological conditions that can be prevented or reversed by adequate supplementation (Fraga 2005). We found that levels of Co, Mn, Fe, Cd and Pb in the pulmonary artery were significantly higher (*P* < 0.001) and those of Mg, Zn and Cu were significantly decreases (*P* < 0.001) in the levosimendan group compared to the control group.

In contrast, trace element deficiency causes cardiomyopathy as a result of the depletion of essential enzymes, which protect the cell membrane from damage by free radicals due to the trace elements playing a key role in essential enzymes such as GPx and SOD. It is therefore not surprising that one of the important biological functions of trace elements is antioxidation. GPx and SOD reduce the production of hydrogen peroxide and superoxide, therefore diminishing the propagation of free radicals (Kosar et al. 2006; Bolli 1988).

These results suggest that short-term levosimendan treatment caused an increase in free radical production and a decrease in antioxidant enzyme activity in the pulmonary artery of levosimendan-treated rats. It also caused a decrease or increase in the levels of many minerals in the pulmonary artery, which is an undesirable condition for normal pharmacological function.
